# Supplementary material for: Ensemble AnalySis with Interpretable Genomic Prediction (EasiGP): Computational tool for interpreting ensembles of genomic prediction models
Source: Plant Genome. 2025 Oct 15;18(4):e70138. doi: 10.1002/tpg2.70138 (PMC12528827; doi:10.1002/tpg2.70138)
Supplement: Supplementary file 1 [file TPG2-18-e70138-s001.docx]

**Supplementary materials**

**Table S1:** The total number of genomic markers (SNPs) and the total number of records obtained from recombinant inbred lines (RILs) scored twice in each population, created from Tomura et al. (2025). “Imputation” indicates the total number of SNPs after excluding SNPs containing more than 10% of missing marker calls during the missing allele imputation process with the most frequent alleles. Subsequently, remaining SNPs were filtered through linkage disequilibrium (LD) with a threshold of 0.8. The total number of final SNPs after the “LD filtering” depends on the combination of RILs included in the training set in each prediction scenario.

| Population name | SNPs | | | | Total  record |
| --- | --- | --- | --- | --- | --- |
|  | Original | | Imputation | LD filtering |  |
| W22TIL01 | 13,089 | 13,042 | | 274 - 322 | 444 |
| W22TIL03 | 16,110 | 16,076 | | 295 - 342 | 540 |
| W22TIL11 | 13,188 | 13,153 | | 268 - 314 | 438 |
| W22TIL14 | 11,396 | 11,375 | | 270 - 320 | 464 |
| W22TIL25 | 14,885 | 14,857 | | 294 - 341 | 618 |

**Figure S1**: Simplified illustrative view of a graph attention network (GAT) in this study. Each genomic marker node was connected to the phenotype node with an edge directed from the marker nodes to the phenotype node. Key predictive information was extracted from each marker node as an embedding in a vector format through convolution, subsequently gathered with weights as attentions to calculate the embedding of the phenotype node. The embedding of each node was reassigned to the corresponding node as the initial node information for the next embedding extraction (hidden) layer. The embedding size was determined by the number of channels in each hidden layer (2 in this example). At the last (outer) layer, the size of the embedding was set as 1. The embedding of the phenotype node at the last layer is returned from GAT as a predicted phenotype.
